# Accelerated functional brain aging in major depressive disorder: evidence from a large scale fMRI analysis of Chinese participants

**DOI:** 10.1038/s41398-022-02162-y

**Published:** 2022-09-21

**Authors:** Yunsong Luo, Wenyu Chen, Jiang Qiu, Tao Jia

**Affiliations:** 1 College of Computer and Information Science, Southwest University, Chongqing, 400715 People’s Republic of China; 2 Faculty of Psychology, Southwest University (SWU), Chongqing, 400715 People’s Republic of China; 3 Key Laboratory of Cognition and Personality (SWU), Ministry of Education, Chongqing, 400715 People’s Republic of China; 4 Southwest University Branch, Collaborative Innovation Center of Assessment Toward Basic Education Quality at Beijing Normal University, Chongqing, 400715 People’s Republic of China

**Keywords:** Depression, Diagnostic markers

## Abstract

Major depressive disorder (MDD) is one of the most common mental health conditions that has been intensively investigated for its association with brain atrophy and mortality. Recent studies suggest that the deviation between the predicted and the chronological age can be a marker of accelerated brain aging to characterize MDD. However, current conclusions are usually drawn based on structural MRI information collected from Caucasian participants. The universality of this biomarker needs to be further validated by subjects with different ethnic/racial backgrounds and by different types of data. Here we make use of the REST-meta-MDD, a large scale resting-state fMRI dataset collected from multiple cohort participants in China. We develop a stacking machine learning model based on 1101 healthy controls, which estimates a subject’s chronological age from fMRI with promising accuracy. The trained model is then applied to 1276 MDD patients from 24 sites. We observe that MDD patients exhibit a +4.43 years (*p* < 0.0001, Cohen’s *d* = 0.31, 95% CI: 2.23–3.88) higher brain-predicted age difference (brain-PAD) compared to controls. In the MDD subgroup, we observe a statistically significant +2.09 years (*p* < 0.05, Cohen’s *d* = 0.134525) brain-PAD in antidepressant users compared to medication-free patients. The statistical relationship observed is further checked by three different machine learning algorithms. The positive brain-PAD observed in participants in China confirms the presence of accelerated brain aging in MDD patients. The utilization of functional brain connectivity for age estimation verifies existing findings from a new dimension.

## Introduction

Global population aging is expected to be one of the prominent social changes of the 21st century [1]. The resulting burden of age-related functional decline and disease would challenge all sectors of society, especially healthcare [2]. Therefore, understanding the biological link between aging and disease risk becomes increasingly important to provide effective care and treatment [3]. Aging can be regarded as a dynamic process in which an individual gradually losses her function as cumulative age-related damage accumulates. The brain structure and function are also significantly changed during this process [4]. As the central nervous system may age dissimilarly to the rest of the body [5], brain-specific aging markers may be of particular importance in assessing the risk of cognitive decline and propensity to neurodegenerative diseases [6].

Accelerated aging of the brain refers to the phenomenon that an individual’s brain appears older compared with the expected chronological age. Brain predicted age difference (brain-PAD), calculated as the difference between the estimated brain age from neuroimaging and the chronological age, is predisposed to be associated with the risk of cognitive aging or age-related brain disorders [7]. This neuroimaging-based biomarker is observed in several neurological disorders [8, 9], including schizophrenia [10–12], Alzheimer’s disease [13–15], epilepsy [16], multiple sclerosis [17], and traumatic brain injury [18]. Furthermore, an association between brain-PAD and mortality [19] has also been reported.

Recent research set out to explore the relationship between accelerated brain aging and major depressive disorder (MDD), a widespread, debilitating, and disabling psychiatric disorder [20] associated with cellular senescence [21, 22] and cognitive decline [23]. Despite the positive association reported [11, 24–29], current studies still present several limitations. First, the size of samples can have a great influence on the stability of the relationship discovered. While subjects with diverse sizes are investigated [8, 30], only one study analyzes data from more than 1000 individuals [31], to the best of our knowledge. Moreover, most of the current studies are based on Caucasian subjects. The generalizability of the association needs to be further verified in subjects from different ethnic and cultural backgrounds. In the meanwhile, the brain age is usually estimated from the structural MRI, with the gray or white matter volume and cortical thickness as the key feature [32]. But the brain age estimated from other types of neuroimaging data is needed for verifications. Finally, current studies often rely on only one machine learning algorithm to estimate brain age. Since different algorithms yield different estimations, it is reasonable to suspect that the conclusion drawn is algorithm sensitive. The statistically significant association originally reported may vanish when a new algorithm is applied.

To cope with these limitations, we make use of the resting-state functional magnetic resonance imaging (rsfMRI) data [33] collected by the REST-meta-MDD [34, 35], which is a coordinated multisite project from China containing over 1000 MDD patients and normal controls. We utilize three different machine learning algorithms to estimate brain age from resting-state functional connectivity [36–38]. We further propose a stacking model to combine results from the three algorithms to reach a more optimal age estimation. We conduct separate analyses on results obtained from each algorithm to check the robustness of the conclusion drawn. We confirm the existence of the positive association between accelerated brain aging and MDD based on subjects in China. The brain-PAD is significantly higher in MDD patients compared to controls and the conclusion is not affected by the machine learning algorithm applied. We separately analyze MDD patients with different depression severity, illness duration, episode status, and medication status to investigate the association between brain-PAD with demographic (age, sex) and clinical characteristics. We find a significant correlation between brain-PAD and illness duration in MDD patients as well as a higher brain-PAD in antidepressant users than in medication-free patients.

## Methods

### Samples

We conduct this study through rsfMRI indices of MDD patients and matched controls (aged 12–82 years) from the REST-meta-MDD consortium, which consists of 25 research groups from 17 hospitals in China. All MDD patients are hospital diagnosed and conducted at least a T1-weighted structural scan and a rsfMRI scan. All subjects agree to provide diagnosis, age, gender, and education years. The 17-item Hamilton Depression Rating Scale (HDRS) is administered to some patients with other tabular data provided, including episode status (if the patient’s prior and current episodes are diagnosed as MDD according to ICD10 or DSM-IV), medication status (whether antidepressants are used), and illness duration. After quality control, we reach a sample set of 1276 MDD patients and 1101 controls from 24 sites. The sample size and scanning parameters for each site are provided in Supplementary Table S1 and Table S2. Written informed consent is signed by participants at each local site, and all data are de-identified and anonymized. Besides, approvals from the local institutional review board and ethics committee are granted at all sites.

### rsfMRI data preprocessing and functional brain network construction

The Data Processing Assistant for Resting State fMRI (DPARSF) [39] is used as a standardized preprocessing pipeline. To obtain functional connectivity, we first extract 116 averaged blood oxygen level-dependent (BOLD) signals based on the Automated Anatomical Labeling (AAL) atlas. Next, we calculate the Pearson correlation coefficients between the BOLD activity time series. We use the Z-Score method [40] to normalize the functional connectivity of each subject to reduce the effect of the imaging sites. We also test the Combat method [41] whose results are presented in Supplementary Information Tables S3 and S4. In general, the Z-Score method performs better in this study. More details of the data preprocessing process are presented in Supplementary Information S2.

### Model training and evaluation

**Fig. 1:**
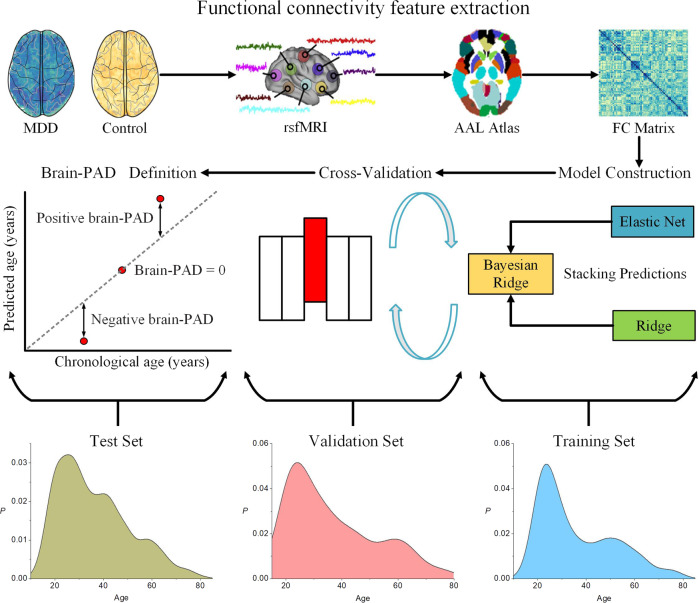
The flow chart of the analysis. Time series for each subject are extracted from rsfMRI according to the AAL atlas. The Pearson correlation is used to get the functional connectivity matrix. Next, the upper triangle of the functional connectivity matrix is stretched into a one-dimensional vector to get each subject’s feature vector. The feature vectors are concatenated to obtain input data matrices. A brain age prediction model is trained and five-fold cross-validated on the training set. Finally, brain-PAD scores are obtained by applying the model in the hold-out validation set and test set.

To obtain the input feature for the model, we reshape the upper triangle of the whole-brain correlation matrix into a one-dimensional vector with 6670 elements. To determine the brain aging pattern in healthy individuals, we first train a brain age prediction model on the training set containing 1101 normal controls. Next, we utilize the model to estimate the brain age of 1276 MDD patients on the test set. The brain age prediction is first carried out by three classical supervised learning algorithms: elastic net [42, 43], bayesian ridge [44], and ridge regression [31, 45, 46]. Furthermore, we introduce a stacking model [36] from ensemble learning [47] to combine results from the three algorithms, which gives the best estimation results. The flow is shown in Fig. 1 and Supplementary Fig. S1. The four models all come to consistent conclusions in subsequent experiments. To avoid switching between different methods and make the flow of the paper more concise, we use the results from the stacking method in the main text. Analyses based on the other three algorithms are included in Supplementary Table S5.

We evaluate our model performance in the control and MDD groups separately. We first evaluate the model on the entire training set with five-fold cross-validation. Then, the same model in each fold is used to predict the brain age of MDD patients on the entire test set. The performance of the four models is evaluated based on the following three metrics: mean absolute error (MAE), mean squared error (MSE), and mean coefficient of determination (*R*^2^). All models are implemented through the Python-based sklearn package with all parameters set as the default value.

### Statistical analyses

To determine whether brain aging is accelerated in MDD patients relative to controls, we split the entire controls to get a fixed training set and a validation set using the hold-out method [48]. While modest in size, this hold-out validation set consists of normal controls from all sites with the entire age span, providing an unbiased age representation of Rest-meta-MDD. As the aim of this study is to explore potential brain age difference between normal controls and MDD patients, we separately estimate the brain age in the two groups. The model is trained and tested in the hold-out validation set composed of normal controls. The trained model is then applied to all MDD patients in the test set to estimate their brain ages. The chronological age is subtracted from the estimated age to get the brain-PAD as the outcome variable for statistical analysis. The five-fold cross-validation is used to compare the overall performance of different models. The hold-out validation set is used as a normal control group for brain-PAD comparison. Due to factors such as regression dilution and non-Gaussian age distribution [49], we need to perform an age-bias correction. We apply a post hoc correction for the residual age effect on the test set [45, 50–53]. Following Peng et al. [54], we train a linear regression model for brain age bias correction. We calculate the regression line between the chronological age and the estimated age on the hold-out validation set. Then the slope and intercept of the regression line are used to adjust brain-predicted age values in the testing set. The steps of this process are shown in Supplementary Information S4. The brain-PAD is independent of chronological age after the age-bias correction (Supplementary Figs. S2 and S3). We apply the univariate generalized linear model (GLM) with gender, diagnosis, age, and age as covariates to explore the relationship between brain-PAD and clinical characteristics [27]. Furthermore, the two-sample t-test is used to compare the brain-PAD in different subgroups. Multiple comparisons are corrected by false discovery rate correction. The threshold for statistical significance is set at *p* < 0.05.

## Results

### Model performance

**Fig. 2:**
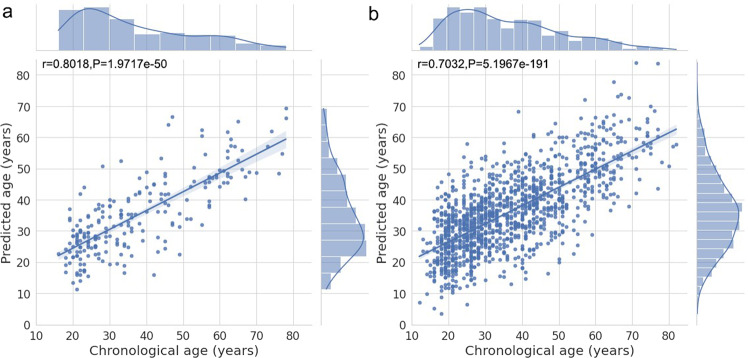
Correlation between the chronological age and predicted age. **a** The predicted age (y-axis)on the validation set. **b** The predicted age on the test set. The lines represent the regression results and the shades correspond to the 95% confidence interval.

The models obtained from each fold of the training set are used to estimate the brain age of individuals for the rest of the controls in the validation set as well as the MDD patients in the test set. Table 1 shows the performance of four models with 882 training subjects, 219 validation subjects, and 1276 test subjects. Among the three classical machine learning algorithms, the bayesian ridge achieves the best performance. But the stacking model with ensemble learning outperforms all of them, giving rise to the lowest MAE and MSE in both the validation and test set. The performance of other widely used models, such as XGBoost [26], SVM [27], and MLP [13] is not as good as the three models applied under default parameters (Supplementary Table S6). The correlation between chronological age and predicted age on the validation set and test set are presented in Fig. 2a, b.

**Table 1:** Performance of four models.

Model	MAE	MSE	R2
Validation set			
Elastic net	8.2327 ± 0.4608	103.7454 ± 10.9086	0.5670 ± 0.0486
Ridge	8.7749 ± 0.5662	127.4526 ± 17.7452	0.4691 ± 0.0665
Bayesian ridge	7.8057 ± 0.4420	97.4546 ± 10.5659	0.5934 ± 0.0440
Stacking	7.7287 ± 0.5547	95.3625 ± 11.8727	0.6026 ± 0.0456
Test set			
Elastic net	8.4156 ± 0.0582	110.2473 ± 1.2672	0.4839 ± 0.0059
Ridge	9.4921 ± 0.2447	143.8590 ± 6.8188	0.3265 ± 0.0319
Bayesian ridge	8.3817 ± 0.0609	110.6582 ± 1.2202	0.4820 ± 0.0057
Stacking	8.3055 ± 0.0535	108.4852 ± 1.2633	0.4837 ± 0.0071

### The relative feature importance for normal controls

**Fig. 3:**
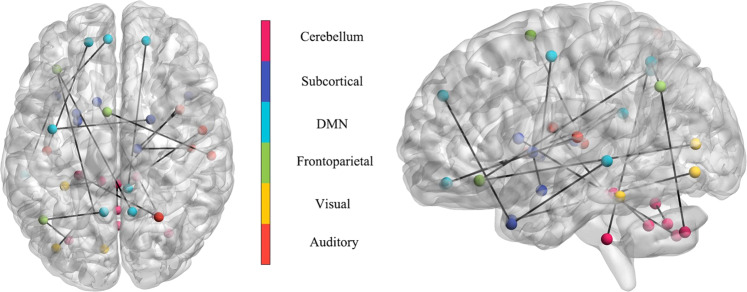
The top 20 most important functional connectivity features obtained from the bayesian ridge model. These features consist of functional connectivity between the cerebellum superior and vermis8, medial superior frontal gyrus and middle temporal gyrus, amygdala and lenticular nucleus putamen, orbital inferior frontal gyrus and precuneus, angular gyrus and precuneus. Statistical values are obtained from Pearson correlations two-sided test.

We calculate the correlation between the functional connectivity features and the chronological age (Supplementary Fig. S4). Among all the total 6670 functional connectivity features, 3196 features show positive correlations with age (mean correlation = 0.0645 ± 0.0495, range (6.1691*e*−05, 0.3017)). 3474 features show negative correlations with age (mean correlation = −0.0691 ± 0.0545, range (−0.3334, 3.7818*e*−05)). In particular, the most positive correlation is found for the precentral gyrus-Heschel gyrus [55, 56]. The most negative correlation is found for the median cingulate and paracingulate gyry-inferior parietal gyrus, excluding supramarginal and angular gyri [57, 58]. In addition, we identify brain regions that the machine learning algorithm considers to be significant in brain age estimation using the feature importance [59]. The feature importance values are normalized to give the top 20 functional connectivity features (Fig. 3). The main brain regions include the cerebellum [60] superior and vermis8, the medial superior frontal gyrus and middle temporal gyrus [61], the amygdala and lenticular nucleus putamen. These brain regions are associated with brain development and atrophy, which are consistent with previous studies.

### Accelerated functional brain aging in MDD

**Fig. 4:**
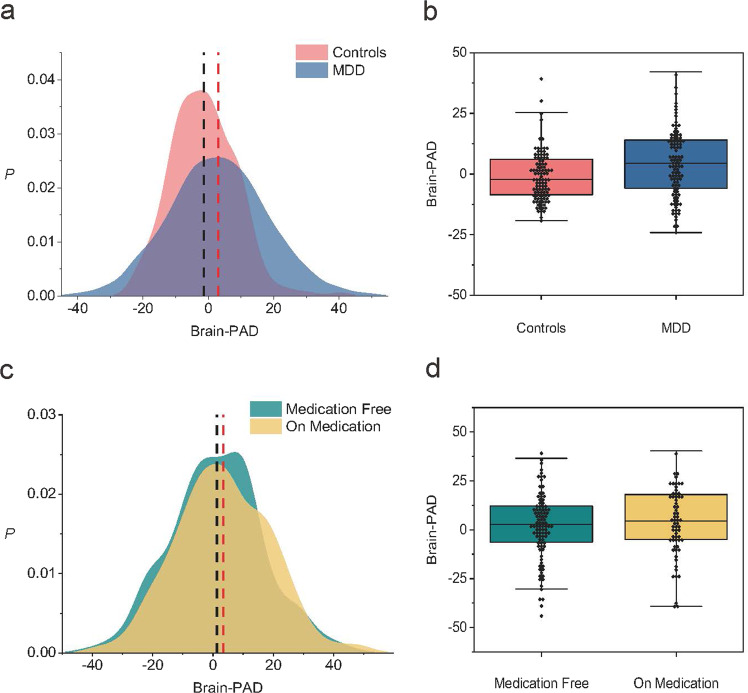
The brain-PAD in different subgroups. **a** The brain-PAD in controls and MDD patients. **b** Group analysis shows that the corrected brain-PAD is significantly higher in MDD patients than in controls (*p* < 0.0001, Cohen’s d = 0.31, 95% CI: 2.23–3.88). **c** The brain-PAD in antidepressant users and medication-free patients. **d** Group analysis shows that the brain-PAD is higher in MDD patients who are taking medication (+3.38 years, 95% CI: 1.86–4.90) than in those medication-free ones (+1.29 years, 95% CI: −0.14–2.72). The difference passes the statistically significant threshold (*p* = 0.0499).

We compare the resulting brain-PAD scores of the MDD patients with the controls in the hold-out validation set to determine whether brain aging is accelerated in MDD patients. Overall, the brain-PAD score before age-bias correction is −1.3731 (SD 9.91) years in the control group and −0.0712 (SD 10.56) years in MDD patients. After applying an age-bias correction procedure, the brain-PAD is +4.43 years higher in MDD patients than in normal controls (*p* < 0.0001, Cohen’s *d* = 0.31, 95% CI: 2.23–3.88), which is shown in Fig. 4a, b. Although different estimations are obtained through different models, results from the other three models all demonstrate a consistent pattern that MDD patients have statistically significant higher brain-PAD scores compared to controls. In addition, GLM shows significant main effects for age (*p* < 0.001), age (*p* < 0.002) and diagnosis (*p* < 0.0001), but not for gender (Table 2A).

**Table 2:** Parameter estimates for all main effects and significant interactions in other clinical characteristics.

	Coef	SE	*z*	*p*	0.025	0.975
(A)						
Intercept	7.64	2.57	2.972	0.003	2.602	12.678
Gender	−0.6052	0.771	−0.785	0.432	−2.116	0.905
Diagnosis	4.7237	1.045	4.519	0	2.675	6.772
Age	−0.4421	0.129	−3.433	0.001	−0.695	−0.19
Age2	0.0046	0.002	3.048	0.002	0.002	0.008
(B)						
Intercept	1.8688	5.087	0.367	0.713	−8.102	11.839
Gender	−1.2863	1.255	−1.025	0.305	−3.746	1.173
Episode	2.2518	1.532	1.47	0.141	−0.75	5.254
Medication	2.9454	1.291	2.282	0.023	0.415	5.475
Age	−0.0285	0.252	−0.113	0.91	−0.522	0.465
Age2	−0.0007	0.003	−0.219	0.827	−0.007	0.006
Education	−0.0022	0.156	−0.014	0.989	−0.307	0.303
Month	0.006	0.012	0.511	0.609	−0.017	0.029

### Brain-PAD comparison for clinical characteristics

To explore the association between brain-PAD scores and clinical characteristics, we use the GLM to fit the brain-PAD of MDD patients with the following explanatory variables: sex, medication status, episode status, education years, and illness duration months (Table 2B). The medication status (*p* = 0.023) has a main effect on the brain-PAD scores of MDD patients. We further apply a two-sample t-test to determine whether the brain-PAD mean value in antidepressant users and medication-free patients are significantly different from each other (Fig. 4c, d). Brain-PAD is +2.09 years higher (*p* = 0.0499, Cohen’s *d* = 0.13452) in antidepressant users than in medication-free ones. Comparisons of other subgroups (sex, episode status) with controls can be found in Supplementary Table S7. While significant differences are observed in all MDD subgroups compared to normal controls, posthoc comparisons of brain-PAD in other clinical characteristics do not demonstrate any significant differences between MDD subgroups except for the medication status. For the two continuous-type clinical characteristics (education years and illness months), we divide the subgroups according to their medians (both are 12 in this study) for brain-PAD comparisons (Supplementary Table S8). Overall, MDD patients with fewer than 12 years of education have a 2.28 years higher brain-PAD than those with greater than or equal to 12 years of education (*p* = 0.00679). Brain-PAD of MDD patients with fewer than 12 months of illness is 1.69 years higher than that in patients with greater than or equal to 12 months of illness. We also calculate correlations between brain-PAD scores and illness months, education years, and HDRS scores separately. Only illness duration is found to be significantly correlated with brain-PAD scores (Spearman R = −0.067, *p* < 0.05, Supplementary Fig. S5).

## Discussion

Biological aging can be defined as a progressive process of decline involving multiple organ systems. While all individuals age chronologically at the same rate, the rate of their biological aging varies from one to the other [62]. Resting-state functional MRI is developed as a common approach to interrogate the myriad of functional systems in the brain without the constraints of any prior assumptions [63]. Machine learning algorithms based on functional connectivity and the availability of large-scale reliable samples allow us to develop generalized models to estimate the brain age of individual subjects [64]. Here, we make use of the Rest-Meta-MDD consortium from China to verify the accelerated brain aging in MDD patients, which is previously observed in Caucasian participants using structural MRI information. We apply four machine learning algorithms based on functional connectivity features to estimate the brain age of individuals with the entire adult lifespan (12–82 years). We observe manifestly accelerated brain aging in 1276 MDD patients. Furthermore, we compare brain-PAD scores between MDD subgroups divided according to clinical characteristics such as medication status and episode status. We confirm that the conclusion drawn in this paper is not algorithm sensitive as results from different algorithms lead to the same conclusion.

Our study benefits from a reliable experimental design. The dataset contains 24 cohorts so the potential site effect is effectively avoided. Instead of using samples from some independent sites as the fixed validation set, we randomly select samples from all sites to constitute the training set and validation set. In this way, the generalizability of the model is improved and the model outcomes are evaluated more objectively [65, 66]. Moreover, we split the normal controls into a fixed training set and a hold-out validation set. We compare the brain-PAD scores of the controls in this hold-out validation set to the MDD patients in the test set. As the validation set is not involved in the development of the brain age prediction model, the risk of overfitting is effectively prevented [67]. The application of four different machine learning algorithms allows us to further validate the consistency of the patterns observed.

The accuracy of our model (Stacking, MAE = 8.3055, *R*^2^ = 0.4837) is better than some previous studies, such as Peter et al. [32] (GPR, MAE = 8.587 years), and Gonneaud et al. [13] (DNN, MAE = 11.90 years). We acknowledge that algorithms used in some other studies yield more accurate age estimation than ours. But multiple factors can affect the performance of the models, such as different features considered and the age distribution of the subjects. The model tends to yield a better prediction using sMRI features than fMRI features [36]. In the meanwhile, the prediction error tends to be smaller when the age distribution of the subjects is narrower. To the best of our knowledge, our work provides a very accurate brain age estimation based on functional connectivity features in a date covering a wide age span (12–82 years).

Although multiple studies are carried out on relatively small samples, conclusions drawn from larger samples tend to be more reliable. First, machine learning algorithms are sensitive to sample size [68–71]. The small size of samples brings a bigger prediction error and a higher risk of overfitting [72]. Moreover, larger samples tend to contain subjects with a wider age distribution. While a wide range of ages makes the estimation challenging, it effectively increases the generalizability of the conclusion [73]. In this study, we make use of the Rest-meta-MDD consortium, which is the largest rsfMRI database of MDD patients. To the best of our knowledge, only one study from ENIGMA uses more subjects than ours, which contains a total of 6989 subjects aged from 18 to 75 years old [31]. But compared with ENIGMA, subjects in Rest-meta-MDD have a bigger life span, ranging from 12 to 82 years old (see the age distribution of the two datasets in Supplementary Figs. S6 and S7). The sufficiently large samples with a wider age span lead to similar conclusions drawn in ENIGMA.

Our results show a +4.43 years gap in terms of the brain-PAD between MDD patients and normal controls (with a Cohen’s *d* effect sizes size of 0.31) at the group level. Compared to previous studies on accelerated brain aging in MDD patients, such as the one by Koutsouleris et al. [11] (+4.0 years, *N* = 104), Han et al. [31] (+1.16 years, *N* = 2675), Dunlop et al. [27] (+2.11 years, *N* = 112), Han et al. [24] (+0.586 years, *N* = 195), our results demonstrate a higher brain-PAD. We speculate that this may be related to the stigmatization of depression in traditional Chinese culture [74]. Some studies suggest that compared to Caucasians, the Chinese tend to deny the existence of depression [75]. Consequently, the level of depression tends to be significantly elevated when MDD is diagnosed [76–78].

We find the significant main effects of age, age, and diagnosis in our regression analysis. In particular, the main effect of medication status on brian-PAD is observed after the inclusion of other clinical characteristics, which is in line with the finding by Sacchet et al. [79]. The comparisons between subgroups of MDD patients show that antidepressant users have a statistically significant higher brain-PAD than medication-free patients (+2.09 years, *p* = 0.0499, Cohen’s *d* = 0.13452). Explanations for this phenomenon are discussed by Han [31]. The antidepressant users are likely to have a more severe or chronic course of the disorder at the time of scanning. Therefore, the larger brain-PAD scores in antidepressant users may be confounded by clinical standards recommending antidepressant use mainly for severe or chronic MDD [80]. In other words, patients with milder symptoms tend not to take antidepressants [81]. To fully understand the adaptation of brain-PAD in response to pharmacotherapy, randomized controlled intervention studies are needed which require more information on the clinical use of antidepressants, such as the dosage and duration. It is also noteworthy that the *p*-value in the study by Han [31] is slightly above 0.05 whereas in our study it is slightly below the threshold of statistical significance. But it is still near the boundary of the threshold line. We honestly report the result, and we also admit that it is far early to draw any conclusion based on this statistic.

Similarly, consistent with the finding obtained by Han et al. [24] using structural brain MRI, we observe a higher brain-PAD in first-episode patients (+4.19 years, *N* = 538) than in recurrent patients (+2.56 years, *N* = 282). We believe the same explanation can be applied. First, as pointed out in previous studies and observed in our work, there is a negative correlation between brain-PAD and illness duration (Spearman R = −0.067, *p* < 0.05). Furthermore, recurrent patients have a longer illness duration than first-episode patients. The median illness duration in recurrent patients (60 months) is 10 times greater than in first-episode patients (6 months) in our data. The corresponding median of brain-PAD score in recurrent patients (0.58 years) is 3.08 years smaller than in first-episode patients (3.66 years). The combination of the two effects gives rise to a higher brain-PAD in first-episode patients than that in recurrent patients. It is implied that there may be a clinically unstable period in first-episode patients. As more treatment is given, patients may become more stable in brain functioning. Hence the brain-PAD decreases with the illness duration [24]. But such a hypothesis needs more clinical information to be further verified through longitudinal studies.

Our results extend the generalizability of accelerated brain aging in MDD patients using the rsfMRI feature of Chinese participants. But several limitations should be considered. Although a standardized preprocessing pipeline is employed at all sites before the aggregation group analysis, some subjects still show measurement bias and missing values in the scan. We address this problem by applying various standardization methods to the features. Although the prediction error is within control, these operations may still bring impact on the final results. Next, multiple brain atlas could be considered to obtain the functional connectivity features. Different functional connectivity will have an impact on the subsequent analysis. Furthermore, different features and models could also have a dramatic effect on the final results. Several studies report the great potential of the multimodal features [82–84] and deep learning algorithms [85–87] in neuroimaging research. More comparisons of neuroimaging features and models are needed in the future to produce more convincing conclusions. Besides, all participants in Rest-meta-MDD are Chinese, the generalizability of our model to other ethnic/racial and cultural backgrounds remained to be explored. Finally, aging is a continuous process, yet few current studies address longitudinal investigations of brain aging, including stage-by-stage analyses of MDD to explore trends in brain-PAD with age to understand the progressive effects of the aging process. More clinical features are still desired in the future to determine the clinical significance of measuring brain-PAD and whether it can be considered as a clinically essential biomarker.

## Supplementary information

Supplementary Information

## Data Availability

Multi-site MDD dataset is available through a reasonable request to the Rest-meta-MDD consortium (http://rfmri.org/REST-meta-MDD). The code of brain age estimation is conditionally available upon request from the corresponding author.
