# Supplementary material for: Accelerated functional brain aging in major depressive disorder: evidence from a large scale fMRI analysis of Chinese participants
Source: Transl Psychiatry. 2022 Sep 21;12:397. doi: 10.1038/s41398-022-02162-y (PMC9492670; doi:10.1038/s41398-022-02162-y)
Supplement: Supplementary file 1 — Supplementary Information [file 41398_2022_2162_MOESM1_ESM.pdf]

Supplementary Information: Accelerated functional brain aging in major depressive disorder: evidence from a large scale fMRI analysis of Chinese participants

Yunsong Luo[1], Wenyu Chen[1], Jiang Qiu[2,3,4] and Tao Jia[1*]

[1*]College of Computer and Information Science, Southwest University, Chongqing, 400715, P. R. China.
[2]School of Psychology, Southwest University (SWU), Chongqing, 400715, P. R. China.
[3]Key Laboratory of Cognition and Personality (SWU), Ministry of Education, Chongqing, 400715, P. R. China.
[4]Southwest University Branch, Collaborative Innovation Center of Assessment Toward Basic Education Quality at Beijing Normal University, Chongqing, 400715, P. R. China.

*Corresponding author(s). E-mail(s): tjia@swu.edu.cn;
Contributing authors: nickolas.luo@gmail.com;
shanyu618@163.com; qiuj318@swu.edu.cn;

S1 Data acquisition

The REST-meta-MDD consortium contains a total of 25 neural cohorts from incoming groups in China. Site 4 (including 24 MDD patients and 24 controls) is excluded in that it is a duplicate of site 14 (detected during the preparation of REST-meta-MDD data for open sharing). We construct a functional connectivity network for each subject based on the AAL atlas using time series data from subjects at the remaining 24 sites. The specific information for each site is shown in Table S1 and Tabele S2. We take the step of adjusting the characteristics of all subjects by various normalization methods. Although these operations may have a slight effect on the final results, we consider these errors to be within reasonable control.

S2 Data preprocessing and quality control

The initial 10 functional images are discarded with the slice acquisition timing discrepancies and the head motion is performed. Linear trends, friston 24 head motion parameters, the white matter signal, and the cerebrospinal fluid signal are regressed out from the functional signal as nuisance covariates. We use the median to fill the vacancy values. Then, we use the Z-score normalization 1 to normalize the functional connectivity of each subject, where μ is the mean and σ is the standard deviation for each functional connectivity feature. The brain age estimation is then performed on the preprocessed functional connectivity data. We have also tested the Combat method. However, the accuracy of models all showed a decrease (Table S3 and Table S4).

$$z = \frac{x - \mu}{\sigma} \tag{1}$$

Our quality control is more relaxed compared to previous studies. We only remove duplicate samples from site 4 and three samples with abnormal age, resulting in 1276 MDD patients and 1101 controls. Various types of subjects are removed in Yan et al. [1] and Yang et al. [2], such as subjects from small sample sites, subjects younger than 18 or older than 65, subjects with vacancies in clinical characteristics, etc. We believe that the age distribution of the training set needs to be made as wide as possible for the model to have better generalizability. At the same time, the proposed algorithm predicts the brain age of subjects based on rsFC features, and the vacancies in clinical features do not affect the algorithm to predict the brain age of subjects based on their rsFC features.

S3 Stacking model

Stacking model is an ensemble learning approach that combines different prediction models in a single model, working at levels or layers. This approach aims to minimize the errors of generalization by reducing the bias of its generalizers. Considering a stacking approach using two levels (level-0 and level-1) in Fig. S1. In level-0, diverse base models are trained, and the prediction of the response variable for each one is performed subsequently. These forecasts are used as a new input feature for the level-1 model, which is also called meta-model [3]. At the same time, the base classifiers need to predict the test set, and the predictions are averaged as a new test set for the meta classifier to predict the desired result.

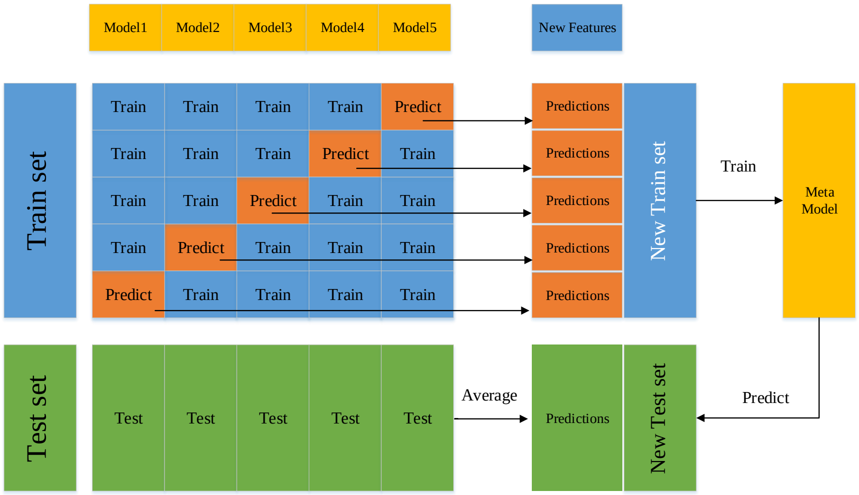

Fig. S1: Schematic diagram of two levels stacking.

S4 Correcting the age dependence of brain-PAD

As shown in Fig. S2a and Fig. S3a, following many existing studies, we observe a significant correlation between brain-PAD and chronological age. It means that the predicted age tends to be biased towards the mean age of the cohort, implying that younger subjects will be predicted to be older and vice versa. To correct for the presence of regression dilution bias in age prediction, we train a linear regression model to fit the predicted age on the validation set with age labels set aside. Then the slope and intercept of this model are used to correct for the predicted age from the test set. The corrected predicted age and the correlation between corrected brain-PAD and chronological age are shown in Fig. S2b and Fig. S3b. Following Peng et al. [4], we define y to be chronological age and x to be the predicted age, and we fit a linear regression (Equation 2) to the hold-out validation set (with labels). The corrected predicted age is estimated by Equation 3.

$$x = ay + b \tag{2}$$

$$\hat{x} = \frac{x - b}{a} \tag{3}$$

This method requires (at the point of estimating a and b from x and y) that the chronological ages are known. For the label-missing (final evaluation) test set, we assume that a and b are generalizable, and use the coefficients previously fit in the hold-out validation set to estimate the brain age.

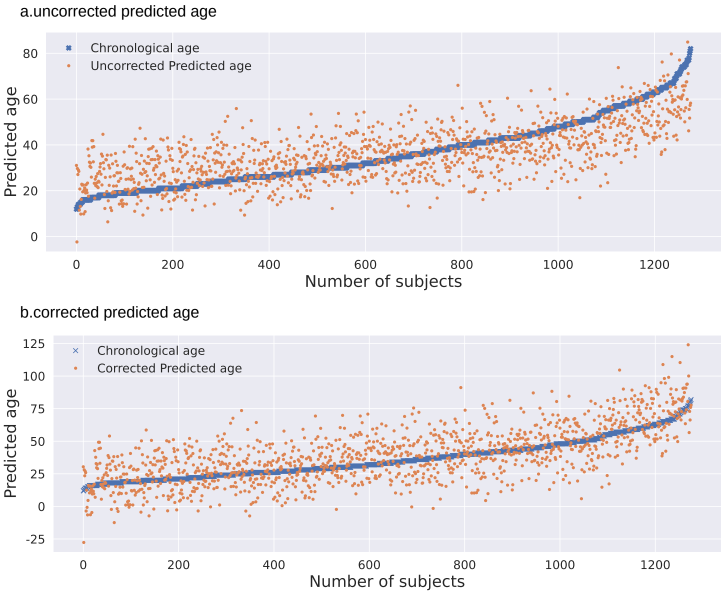

Fig. S2: Comparison of predicted age before and after correction.

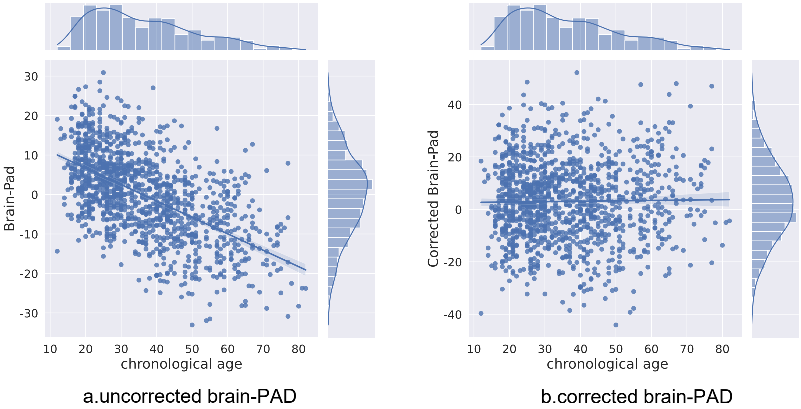

Fig. S3: Correlation between bran-PAD and chronological age.

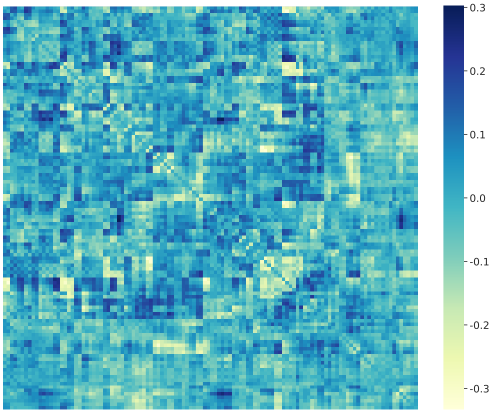

Fig. S4: Correlation between the functional connectivity features and the chronological age. Among all the total 6670 functional connectivity features, 3196 features show positive correlations with age mean correlation $= 0.0645 \pm 0.0495$, range$(6.1691e-05, 0.3017)$. 3474 features show negative correlations with age mean correlation $= -0.0691 \pm 0.0545$ with range$(-0.3334, 3.7818e-05)$.

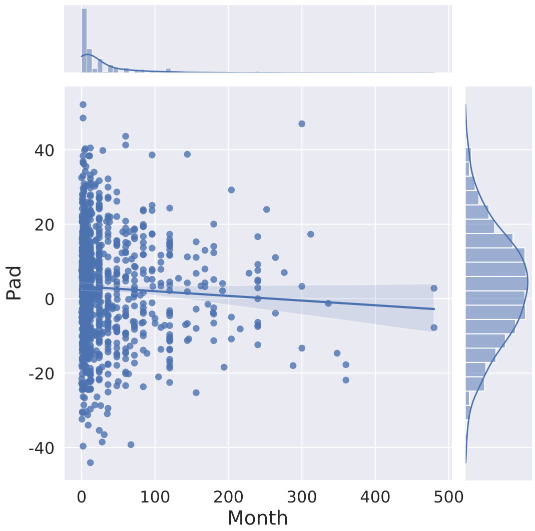

Fig. S5: The correlation between brain-PAD scores and illness duration months in patients with depression. There is a negative correlation between the brain-PAD and illness duration (Spearman $R = -0.067$, $p = 0.03$).

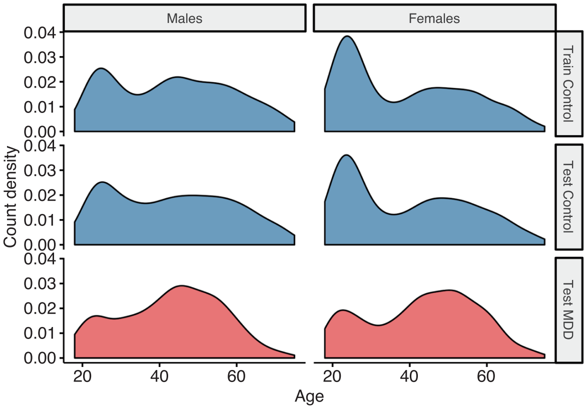

Fig. S6: Age distribution in ENIGMA. The ENIGMA MDD Working Group contains 6989 participants (18-75 years old).

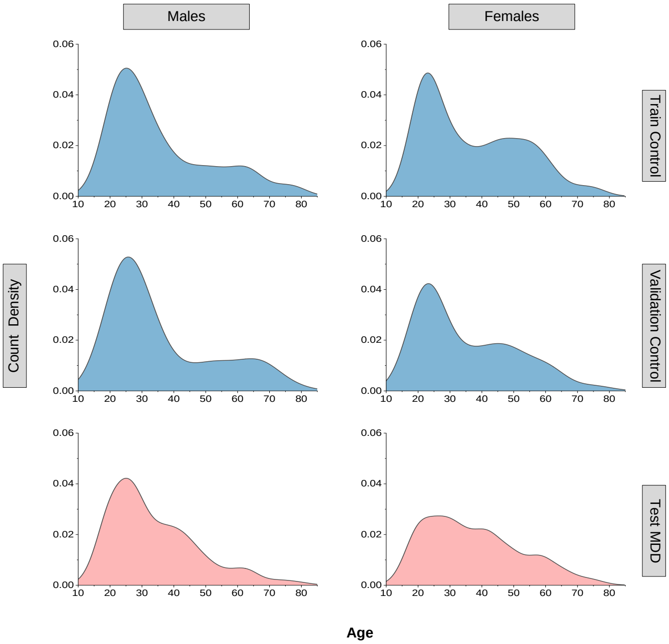

Fig. S7: Age distribution in Rest-meta-MDD. The Rest-meta-MDD contains 2377 participants (12-82 years old).

Table S1: Samples of selected sites.

Serial number	Research groups	MDD patients (n)	NCs (n)
1	National Clinical Research Center for Mental Disorders, Peking University	74	74
2	The Affiliated Guangji Hospital of Soochow University	30	30
3	The Second Xiangya Hospital of Central South University	27	37
5	Department of Psychiatry,Shanghai Jiao Tong University School of Medicin	13	11
6	Department of Psychiatry,Shanghai Jiao Tong University School of Medicine	15	15
7	Sir Run Run Shaw Hospital,Zhejiang University School of Medicine	38	49
8	Department of Psychiatry,First Affiliated Hospital,China Medical University	75	75
9	The First Affiliated Hospital of Jinan University	50	50
10	First Hospital of Shanxi Medical University	50	33
11	Department of Psychiatry,The First Affiliated Hospital of Chongqing Medical University	32	29
12	Department of Psychiatry,The First Affiliated Hospital of Chongqing Medical University	32	6
13	The First Affiliated Hospital of Xi an Jiaotong University,Xian Central Hospital	25	17
14	The Second Xiangya Hospital of Central South University	64	32
15	Zhongda Hospital, School of Medicine, Southeast University	50	50
16	Huaxi MR Research Center, West China Hospital of Sichuan University	31	31
17	Department of Psychiatry,The First Affiliated Hospital of Chongqing Medical University	47	44
18	The First Affiliated Hospital, College of Medicine, Zhejiang University	21	20
19	Anhui Medical University	51	36
20	Faculty of Psychology, Southwest University	282	251
21	Beijing Anding Hospital, Capital Medical University	86	70
22	The Institute of Mental Health,Second Xiangya Hospital of Central South University	30	20
23	Mental Health Center, West China Hospital, Sichuan University	32	30
24	First Affiliated Hospital of Kunming Medical University	32	31
25	Department of Neurology,Affiliated ZhongDa Hospital of Southeast University	89	63

Table S2: Data acquisition parameters of selected sites.

Serial number	Scanner	Coil	TR (ms)	TE (ms)	Flip angle	Thickness/gap	Slice number	Timepoints
1	Siemens Tim Trio 3T	32	2000	30	90	4.0 mm/0.8 mm	30	210
2	Philips Achieva 3T	8	2000	30	90	4.0 mm/0 mm	37	200
3	Siemens 1.5 T	16	2000	40	90	5.0mm/1.25mm	26	150
5	GE Signa 3T	32	3000	30	90	5.0mm/0 mm	22	100
6	Siemens Tim Trio 3T	32	2000	30	70	4mm/0mm	33	180
7	GE discovery MR750	8	2000	30	90	3.2 mm/0 mm	37	184
8	GE Signa 3T	8	2000	30	90	3.0 mm/0 mm	35	200
9	GE Discovery MR750 3.0T	8	2000	25	90	3.0 mm/1.0 mm	35	200
10	Siemens Tim Trio 3T	32	2000	30	90	3.0 mm/1.52 mm	32	212
11	GE Signa 3T	8	2000	30	90	5 mm	33	200
12	GE Signa 3T	8	2000	30	90	5 mm	33	240
13	GE Excite 1.5T	16	2500	35	90	4 mm/0 mm	36	150
14	Siemens Tim Trio 3T	32	2500	25	90	3.5 mm/0 mm	39	200
15	Siemens Verio 3.0T MRI	12	2000	25	90	4 mm/0 mm	36	240
16	GE Signa 3T	8	2000	30	90	5mm/0mm	30	200
17	GE Signa 3T	8	2000	40	90	4.0 mm/0 mm	33	240
18	Philips Achieva 3.0 T scanner	8	2000	35	90	5.0/1.0 mm	24	200
19	GE Signa 3T	8	2000	22.5	30	4.0 mm/0.6 mm	33	240
20	Siemens Tim Trio 3T	12	2000	30	90	3.0 mm/1.0 mm	32	242
21	Siemens Tim Trio 3T	32	2000	30	90	3.5 mm/0.7 mm	33	240
22	Philips Gyroscan Achieva 3.0T	32	2000	30	90	4.0 mm/0 mm	36	250
23	Philips Achieva 3.0T TX	8	2000	30	90	4.0 mm/0 mm	38	240
24	GE Signa 1.5T	8	2000	40	90	5/1mm	24	160
25	Siemens Verio 3T	12	2000	25	90	4.0mm/0 mm	36	240

Table S3: Comparison of models for Combat and Z-score (validation set)

Validation set		
Model	MAE (Combat)	MAE (Z-score)
Elastic Net	13.5363 ± 4.6699	8.2327 ± 0.4608
Ridge	12.9766 ± 3.6070	8.7749 ± 0.5662
Bayesian Ridge	12.1408 ± 4.2740	7.8057 ± 0.4420
Stacking	11.0202 ± 4.5119	7.7287 ± 0.5547

Table S4: Comparison of models for Combat and Z-score (test set)

Test set		
Model	MAE (Combat)	MAE (Z-score)
Elastic Net	12.1243 ± 0.0916	8.4156 ± 0.0582
Ridge	16.1888 ± 0.7375	9.4921 ± 0.2447
Bayesian Ridge	12.8572 ± 0.3426	8.3817 ± 0.0609
Stacking	11.2833 ± 0.1501	8.3055 ± 0.0535

Table S5: Comparison of brain-PAD in different models.

Model	MDD-NC(Mean-PAD)	*P* value	Cohen's *d*
SVM-linear	5.215	4.09e-09	0.32
EN	3.282	5.88e-05	0.23
Ridge	5.186	5.22e-09	0.32
BR	4.532	3.55e-08	0.31

Table S6: Performance of different models.

Validation set			
Model	MAE	MSE	R^2
SVM-linear	8.9308 ± 0.6309	132.4733 ± 20.3135	0.4482 ± 0.0771
SVM-RBF	11.6761 ± 0.8564	231.1530 ± 30.6658	0.04127 ± 0.0647
RandomForest	15.1180 ± 1.3921	433.7388 ± 57.8672	-0.8006 ± 0.1541
MLP	12.3220 ± 0.5293	247.6958 ± 20.3218	-0.1595 ± 0.0951
XGBoost	23.3025 ± 1.8482	776.5373 ± 110.7799	-2.2516 ± 0.5404
Test set			
SVM-linear	9.6537 ± 0.3379	148.42189 ± 9.4821	0.3051 ± 0.0443
SVM-RBF	11.0753 ± 0.0464	205.1567 ± 3.4306	0.0395 ± 0.0160
RandomForest	15.4476 ± 0.1794	408.0862 ± 8.6558	-0.9104 ± 0.0405
MLP	13.2508 ± 4.1303	278.9408 ± 167.5347	-0.5317 ± 0.4558
XGBoost	34.2420 ± 0.1923	1590.5002 ± 22.9607	-6.4457 ± 0.1074

Table S7: Comparison of brain-PAD between different clinical characteristics.

MDD vs NC	Number	Brain-PAD	*P* value	SD	Cohen's *d*	95% CI
MDD	1276	4.43	3.49e-08	15	0.31	2.23-3.88
First-episode MDD	538	4.19	7.80e-06	14.99	0.31	1.54-4.08
Recurrent episode	282	2.56	2.10e-02	14.85	0.20	-0.55-2.93
antidepressant users	408	4.75	3.99e-06	15.62	0.34	1.85-4.90
Medication free	447	2.66	7.16e-03	15.41	0.19	-0.14-2.72
Male	463	5.07	1.85e-07	14.89	0.37	2.34-5.05
Female	813	4.06	2.30e-06	15.06	0.29	1.65-3.73

Table S8: Comparison of brain-PAD in education years and illness duration months.

MDD subgroup	Number	Brain-PAD
Education<12	634	4.2
Education>=12	642	1.92
Duration <6	329	4.05
Duration <12	461	3.7
6<= Duration <12	132	2.83
12<= Duration <24	130	2.48
Duration >=12	524	2.01
Duration >=24	394	1.86

References

[1] Yan, C.-G., Chen, X., Li, L., Castellanos, F.X., Bai, T.-J., Bo, Q.-J., Cao, J., Chen, G.-M., Chen, N.-X., Chen, W., *et al.*: Reduced default mode network functional connectivity in patients with recurrent major depressive disorder. Proceedings of the National Academy of Sciences **116**(18), 9078–9083 (2019)

[2] Yang, H., Chen, X., Chen, Z.-B., Li, L., Li, X.-Y., Castellanos, F.X., Bai, T.-J., Bo, Q.-J., Cao, J., Chang, Z.-K., et al.: Disrupted intrinsic functional brain topology in patients with major depressive disorder. Molecular psychiatry, 1–9 (2021)

[3] Shamaei, E., Kaedi, M.: Suspended sediment concentration estimation by stacking the genetic programming and neuro-fuzzy predictions. Applied Soft Computing **45**, 187–196 (2016)

[4] Peng, H., Gong, W., Beckmann, C.F., Vedaldi, A., Smith, S.M.: Accurate brain age prediction with lightweight deep neural networks. Medical image analysis **68**, 101871 (2021)
